# Systematic Literature Review of Realistic Simulators Applied in Educational Robotics Context

**DOI:** 10.3390/s21124031

**Published:** 2021-06-11

**Authors:** Caio Camargo, José Gonçalves, Miguel Á. Conde, Francisco J. Rodríguez-Sedano, Paulo Costa, Francisco J. García-Peñalvo

**Affiliations:** 1 Instituto Politécnico de Bragança, 5300-253 Bragança, Portugal; caioo.rafael@gmail.com (C.C.); goncalves@ipb.pt (J.G.); 2 CeDRI—Research Centre in Digitalization and Intelligent Robotics, 5300-253 Bragança, Portugal; 3 INESC TEC—Institute for Systems and Computer Engineering, 4200-465 Porto, Portugal; paco@fe.up.pt; 4 Robotics Group, Engineering School, University of León, Campus de Vegazana s/n, 24071 León, Spain; francisco.sedano@unileon.es; 5 Universidade do Porto, 4200-465 Porto, Portugal; 6 GRIAL Research Group, Computer Science Department, University of Salamanca, 37008 Salamanca, Spain; fgarcia@usal.es

**Keywords:** robotics, education, realistic simulators, sensors, actuators, physics engine

## Abstract

This paper presents a systematic literature review (SLR) about realistic simulators that can be applied in an educational robotics context. These simulators must include the simulation of actuators and sensors, the ability to simulate robots and their environment. During this systematic review of the literature, 559 articles were extracted from six different databases using the *Population, Intervention, Comparison, Outcomes, Context* (PICOC) method. After the selection process, 50 selected articles were included in this review. Several simulators were found and their features were also analyzed. As a result of this process, four realistic simulators were applied in the review’s referred context for two main reasons. The first reason is that these simulators have high fidelity in the robots’ visual modeling due to the 3D rendering engines and the second reason is because they apply physics engines, allowing the robot’s interaction with the environment.

## 1. Introduction

With the development of computers, simulation has become a powerful tool in the many areas in which it can support design, planning, analysis and decision-making in research and development [1,2,3,4,5].

Simulation is the process of designing a model of an actual or theoretical physical system, executing the model and analyzing the output. It helps to understand our reality and its complexity by building artificial objects and dynamically acting out roles. The simulation application enables learning about something in a very effective way and, by modifying environment rules, we can observe the results of the interactions. It is also an interdisciplinary field, applied in all research fields in society, from engineering and computer science to economics and social science, and at all different scientific study levels, even to manufacturers. Researchers and companies may build experimental systems using simulators even in the early development stages, testing complexity, reality and specificity. The simulation tests can be gradually increased to a level where these virtual systems can help to solve real challenges of the physical world, create new revolutionary products and push human imagination and creative boundaries—one of the main applications of simulation in the robotics field. By designing new products and investigating performance, simulation permits the study of structures, characteristics and a robotic system’s function no matter how complex it is. Although, as the system’s complexity increases, the need for simulation rises at the same level. Hence, the simulation tools can, for sure, improve design, development and robotic operating systems. Simulators utilizing a graphical user interface and visualization tools can provide us with a realistic way of visualizing the robotics system’s operation [1].

Robot simulation started to become feasible and got more attention when the computational power of personal computers increased over the years in a significant way. In almost every computer today, it is possible to run complex algorithms and many graphical calculations. With that, realistic simulations are also possible thanks to the game industry’s efforts to create realistic visualisation in computer games. The creation of virtual worlds requires considerable processing power to render graphical environments and physics calculations. Consequently, this effort developed software engines that provide high-quality physics simulations and rendering software in the robotics domain [2].

In this context, physics engines are software that allow computers to create physics phenomena that we experience in the real world, that is, rigid body dynamics, collision detection, soft body dynamics, fluid dynamics and other physical aspects, and apply them to 3D objects in games (the most usual application) and other 3D renderings, which affects how those objects interact in the digital world. Game developers and video effects artists use physics engines to create lifelike computer-generated environments for video games, movies and television. Some architects may use physics engines to create realistic 3D renderings for concept designs. Even if a 3D environment does not require real-life physics, a physics engine will allow the designer to customise physics to fit their needs [6,7].Without something like a physics engine telling many different 3D objects how to interact, programming an environment would be extremely time-consuming. Some environments may have hundreds of objects that all interact with each other in various ways. For example, an object in a bowl on a table is interacting with the bowl, the other objects in the bowl, the table and the ground the table sits on. As a game developer or video effects artist, a physics engine will be part of the suite of tools applied to create 3D environments. In many cases, physics engines are included in game engines, 3D modeling suites and 3D rendering tools. However, it may be offered as a standalone or as a plug-in to another software [8,9].

To qualify as a physics engine, a software must:

1. Simulate a variety of physical systems (rigid body dynamics, soft body dynamics, fluid dynamics, etc.);
2. Apply those systems to 3D objects and environments;
3. Work in tandem with other software systems to create a cohesive experience.

The main objective of this work is to present a systematic literature review that allows us to understand whether there are any realistic simulators that are or can be applied in an educational robotics context, and to obtain scientific databases in order to analyze and compare the features of these kinds of simulators. The reason for exploring the educational context is because of the multiple advantages for pre-university students of robotics application [10], specially for developing STEAM related competences [11]. Still within the context of educational robotics, this research seeks to find, analyze and compare realistic simulators capable of simulating robots, sensors and actuators in general. In order to answer the research question of this work and fulfill the goal, this review becomes important for future applications and frameworks that can be developed using these simulation tools to be applied at all educational levels and, as a consequence, in teaching robotics and computer science topics.

The structure of this work is as follows: Section 2 describes all the methodology followed to execute the systematic literature review [12], the research question, the PICOC method and the search string equation that was applied to the databases. Section 3 presents a Preferred Reporting Items for Systematic Reviews and Meta-Analyses (PRISMA) flow diagram with all the papers obtained from the database searches of the previous section. Section 4 discusses and analyzes the results from the selected and relevant papers, after filtering by selection criteria. Finally, in Section 5, conclusions and future work are proposed.

## 2. Method

This paper was conducted by following the systematic literature review methodology presented by Kitchenham [13,14,15]. A systematic literature review is a means of evaluating and interpreting all available research, relevant to a particular research question, topic area, or phenomenon of interest. The SLR aims to present a fair evaluation of a research topic using a trustworthy, rigorous and auditable methodology. The guidelines for conducting an SLR are divided into three phases: planning the review, conducting the review and reporting the review [16,17,18,19,20,21,22].

Before starting the planning of the SLR, a preliminary search is needed on a database, such as Google Scholar, to verify if there is an SLR with the same theme of research. If there is an SLR with the same topic, there would not be any need to conduct a new one [23,24]. In the case of this systematic review of the literature, no results were found, covering the realistic simulators subject, therefore, the SLR can be carried out as new research.

### 2.1. Planning the Review

The first part is the review planning, consisting of the process of identification and definition of the review execution, ensuring that the review is traceable [25]. At the beginning, it is necessary to clearly specify the research question that it aims to investigate. For this work, taking into account the described context in the Introduction, the research questions (RQs) are:

- **RQ1:** In the context of educational robotics, are there any realistic simulators capable of simulating any robot prototype?
- **RQ2:** Are these simulators capable of simulating the robot’s sensors and/or actuators?
- **RQ3:** Is such simulation based on physics engines?

Once the research questions have been defined, the PICOC method proposed by Petticrew and Roberts [22] was followed to define the review scope.

- **Population (P):** Robotics Simulators;
- **Intervention (I):** Realistic Robotics Simulators;
- **Comparison (C):** Compare the already existing robotics simulators;
- **Outcome (O):** Understand the ways of simulate realistic robots, being able to simulate micro-controllers, sensors and actuators as well;
- **Context (C):** Educational Robotics.

### 2.2. Inclusion and Exclusion Criteria

With the PICOC established, the scope of the review has been set, accompanied by the research questions and selection criteria—inclusion (IC) and exclusion (EC)—are defined to select the relevant papers that answer the research questions. For a paper to be selected, it has to meet all the Inclusion Criteria, and if it meets any Exclusion Criteria, it will be excluded.

- **IC1:** The papers are written in English; (AND)
- **IC2:** The papers are reported in peer reviewed conferences or journals or technical reports; (AND)
- **IC3:** The papers that use any kind of simulator, OR simulate realistic robotics, OR simulate sensors OR Actuators.

The Exclusion Criteria are the opposite of the Inclusion Criteria.

- **EC1:** The papers are NOT written in English; (OR)
- **EC2:** The papers are NOT reported in peer reviewed conferences or journals or technical reports; (OR)
- **EC3:** The papers that do NOT use any kind of simulator, OR simulate realistic robotics, OR simulate sensors OR Actuators.

These selection criteria will determine whether, from reading the paper’s title and abstract, it will be included in the review or not, and whether it is useful to include relevant works in the review in terms of its scope.

### 2.3. Search Methodology

The methodology of an SLR differs from a search made randomly on the Internet in several aspects. One of the most relevant is the need to determine the data sources, which should be the most important databases in terms of the research context. The electronic databases used in this work were: ACM Digital Library, IEEE Digital Library, ISI Web of Science, ScienceDirect, Scopus and Springer Link.

These databases were selected for three main reasons:

1. They are well-known databases in this research field;
2. They are relevant databases in the research theme of this literature review;
3. It is possible to use a search string as well as Boolean operators to improve the results of the search process.

Given this procedure, the next step is to define the search string equation for the different databases. It was built using relevant terms from the PICOC methodology and they were connected by Boolean “AND” and “OR” operators [26,27]. Moreover, the asterisk sign operator was used to include both the singular and plural of each term. Taking this into account, the search string equation is shown as follows:

(“educational robotics” OR “educative robotics” OR “robotics and education”) AND (“realistic simulators” OR “prototype” OR “prototyping”)

The search string equation is divided into two main parts. The first part contains three related concepts, which are: “Educational Robotics”, “Educative Robotics” or “Robotics and Education”. These concepts are inclusive and connected between each other, and they were retrieved from the Context from the PICOC methodology. The search string equation will be executed in the all electronic databases in order to gather all the published papers connected with those areas.

The second part of the search equation is related to the main objective of this work, the terms: “Realistic Simulators”, “Prototype” or “Prototyping”. The “Realistic Simulators” term has the role of finding in the electronic databases all the papers that in some way have used a realistic simulator. The two last terms “Prototype” or “Prototyping” are related and help to expand our search, because these words represent one of the main applications for simulators, that is, prototype simulation.

The following describes and shows the search strings equation applied to each database.

1. **ACM Digital Library:** For the ACM Digital Library (http://portal.acm.org, accessed on 8 June 2021) the Advanced Search resource was used, where the search equation was split into two parts placed in two separated search fields; the query syntax returned from this database is is shown below:
  [[All: “educational robotics”] OR [All: “educative robotics”] OR [All: “robotics and education”]] AND [[All: “realistic simulators”] OR [All: “prototype”] OR [All: “prototyping”]]
2. **IEEE Digital Library:** In the IEEE Digital Library (http://ieeexplore.ieee.org, accessed on 8 June 2021), we used the simple search bar on the web site, pasting the search strings there.
3. **ISI Web of Science:** In the ISI Web of Science (http://www.isiknowledge.com, accessed on 8 June 2021) he query terms were posted in the basic search tab to obtain the papers.
4. **ScienceDirect:** For ScienceDirect (http://www.sciencedirect.com, accessed on 8 June 2021), the use of the website was very straight forward; the equation was pasted in the search field to obtain the results from the database.
5. **Scopus:** In the Scopus database (http://www.scopus.com, accessed on 8 June 2021), the use of advanced search was needed in order to obtain the maximum possible results. The entered query strings used were:
  ALL(“Educational Robotics” OR “Educative Robotics” OR “Robotics and Education”) AND ALL(“Realistic Simulators” OR “Prototype” OR “Prototyping”).
6. **Springer Link:** For the Springer Link database (http://link.springer.com, accessed on 8 June 2021), the query string was used in the simple search bar on the website.

### 2.4. Quality Criteria

After the first preliminary part of paper selection, described as the Inclusion and Exclusion criteria, a new set of questions was defined to check the work’s quality before including them in the final literature review.

Each question can be answered with a possible weight between three values: 4.0 (Yes, it answers the question fully), 2.0 (Yes, it answers the question partially) and 0.0 (No, it does not answer the question). These values are assigned to the papers by reading them fully. The quality assessment checklist is shown in Table 1.

**Figure 1:**
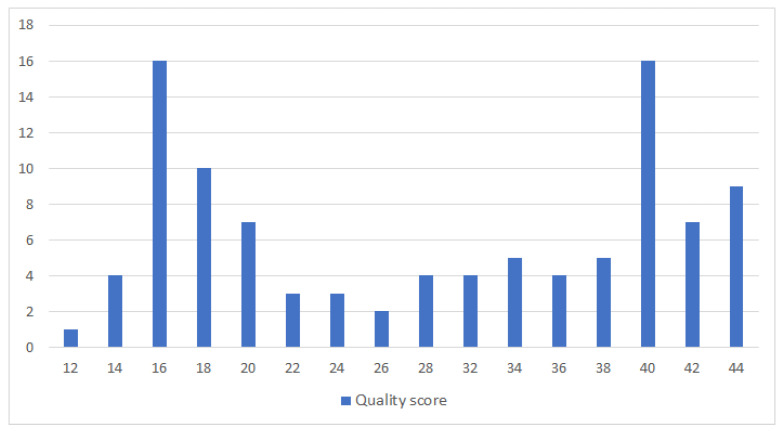
Distribution of Quality Data.

Therefore, each paper can be assigned a maximum of 44.0 points based on the quality criteria. In Figure 1, it can be observed the distribution of these quality data.

The median overall score (out of 44) of the 100 included studies was 30, and the mean overall score was 29.08. We, therefore, decided to set a cut-off score of 30 points. All those papers that exceeded this score were included in the final synthesis.

#### Data Extraction Form

When the quality assessment process of the papers was running out, a data extraction form was made with a set of questions to evaluate the simulators used during the reading of the works. These questions are shown in Table 2.

For the first three questions (DQ1, DQ2 and DQ3), the answer is Boolean so it could be answered with “Yes” or “No”. The following three questions (DQ4, DQ5 and DQ6) should be answered with strings. DQ4 describes the method used in the paper for using the simulator; DQ5 describes whether the simulation was made under a mathematical–physical model; and DQ6 states the name of the simulator applied. The last two questions required selecting one possible option. DQ7 asks if the simulator is free to use, is fully paid or a mix of both and DQ8 asks if the simulator is an open-source platform or not. The result of this data extraction will be presented in the results section in a Table format, in which every mentioned simulator in the papers has a score equal to or above 30.0.

## 3. Results

**Figure 2:**
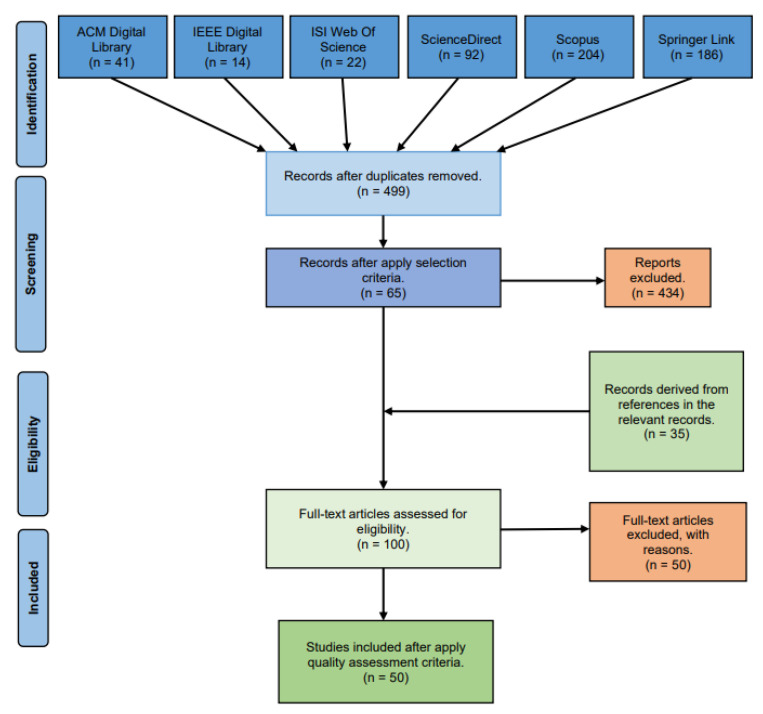
The Systematic Literature Review process. Adapted from [29].

This section presents all the results obtained from the searches on the databases. The data compilation was divided into different phases according to the PRISMA flow diagram, shown in Figure 2, which details the actions taken during the SLR process [28,29].

**Figure 3:**
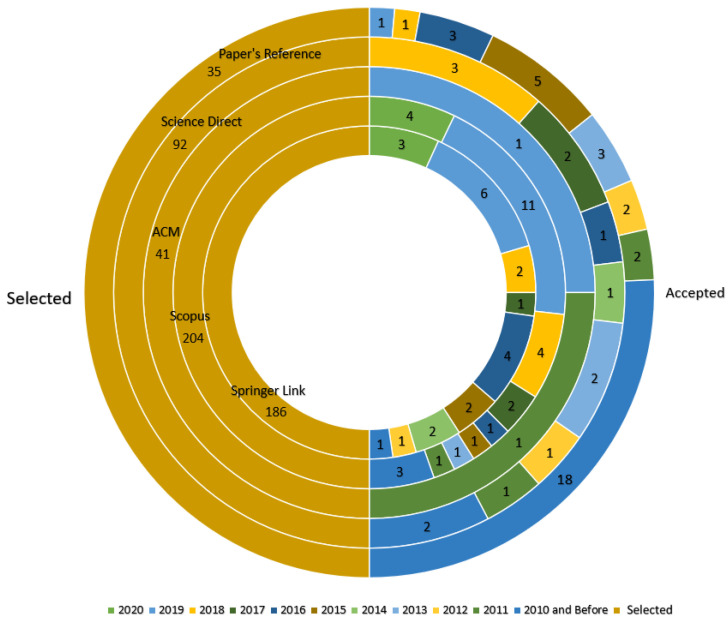
Distribution of publication per year and source.

This process was carried out following the methodology described in Section 2.3. The search on the databases was performed (on 27 August 2020), carrying on with the paper selection process:

1. First, the results retrieved from the initial search were 559 papers in total, distributed in 41 citations from the ACM Digital Library, 14 from the IEEE Digital Library, 22 papers from ISI Web of Science, 92 works from ScienceDirect, 204 citations from Scopus and 186 from Springer Link.
2. After the search, all these references were uploaded and organized into the Parsifal (https://parsif.al/, accessed on 8 June 2021) (the main tool applied to conduct this SLR) and it detected 60 duplicated records that were consequently removed.
3. As result, 499 works were retrieved from the previous step and they were analyzed through the reading of their titles, keywords and abstracts and applying the Inclusion and Exclusion Criteria. From this process, 434 articles were excluded because they did not meet the requirements, leading us to the next phase, with 65 papers.
4. The accepted papers were read in detail. When each article was read, it was scored regarding its quality, applying the quality assessment questions described in Section 2.4. In addition, while reading these works, their references were carefully checked in order to find new articles (as an alternative source) that could address the research question, resulting in 35 new reports (on 7 December 2020). Figure 3 shows the amount of obtained articles per source and year, and those which were accepted per source. A relevant issue to take into account is that none of the selected papers were from the Web of Science or the IEEE Digital library data sources, so these databases are not shown in the graphic.
5. After the evaluation of the papers’ quality, 15 papers that scored higher than or equal to 30 were selected, adding to them the 35 obtained from the reference checking of the previous phase. This resulted in a total of 50 selected works with which to compose the present review, which can be seen distributed by publication year and source in the Figure.

## 4. Discussion and Results

This section describes the results of the developed systematic literature review. How every simulator addresses the research questions (**RQ1, RQ2 and RQ3**) made in the Section 2.1 is discussed, through the data extraction form presented in Table 2. Taking this into account, further subsections point out the answers to the data questions by discussing three main issues: (1) the features of the simulators found in the review; (2) some interesting exceptions about papers included in the study; and (3) the features of the engines, on which the simulators are based. Finally, a subsection describes the Robot Operating System, which is an alternative method for writing the robots’ software in the simulators.

Table 3 presents all the papers selected, showing their quality scores, the name of the simulator used and its ability to simulate robots, and its sensors and actuators. Note that the columns tagged as **d**, **e** and **f** address the answers to Data Questions 1, 2 and 3, meaning whether the simulator is able to simulate robotics, sensors and actuators, respectively.

As shown in Table 3, several simulators were found, and the most used from the selected papers were Sim-Two (22 times), Gazebo (7 times) and V-REP (4 times). Something to point out is that the papers with a score of 42 points or 44 points are those applied in the educational context that were able to simulate all the features inquired for the research questions, but with a few exceptions (such as **Exception 1** and **Exception 2**) that are described in the next subsections.

Although the most used simulators were Sim-Two, Gazebo and V-REP, others were found through the reading process (Table 3). The table presents a distribution of the simulators in this literature review.

As can be noticed, the most mentioned simulators found in the full-read process were: USARSim, Gazebo, Webots, Sim-Two, Stage/Player, V-REP, UberSim, MuRoSimF and the Microsoft Robotics Studio. To better understand the reason why the papers’ authors cite or use them, each simulator is investigated and described in the next subsection.

### 4.1. Simulators Features

In this subsection the features of the simulators found in the literature are described. Besides the papers studied during the research methodology, in this part it another search was made in Google Scholar in order to find papers that contain the simulators’ details and facts to add and support the information about the simulators presented in Table 4. This is necessary because, as the analysis in Figure 3 shows, most of the papers included are from before 2016, and many of the features of the simulators may have changed through the years due to technology evolution. The simulator features can be found in Table 5.

As can be observed, many simulators were found during the search. The main characteristic among some of them is that they are based on physics engines, which allows them to simulate the robot and the robot’s environment in a more realistic way. Another important feature that was noticed was that some of these simulators are defined with multiple simulation purposes (ie V-REP, Webots, Gazebo, SimTwo and others), which means they are able to simulate several types of robots, unlike others that only simulate one type of robot (i.e., ARGoS, RoSoS, UberSim, OpenHRP3, Khepera and others).

As a result of searching information about the simulators listed above, an Excel file was uploaded into a GitHub repository, a summarized Table showing only the simulator’s main features. The repository’s link can be found in Appendix A.

Finally, most of the simulators were developed in the 2000 s, and it can be observed that the most mentioned simulators in Table 5 still continue to have updates for current technologies since their launch. An exception to these simulators is the Microsoft Robotics Studio, which has been discontinued, as have UberSim and Khepera.

### 4.2. Exception Points

This subsection describes some simulators and papers that were considered exceptions, found during the research of this SLR. Although they do not completely fulfill the previously defined requisites, they represent relevant work that it is worth to mention.

1. The first exception point to be discussed in the Table 3 is the **Exception 1** found in the paper [61]. This paper has as its title: “Mathematical modelling, simulation and experimental verification of a Scara robot”, from Das, M. T., & Dülger, L. C. The authors developed a complete mathematical model of the Scara robot (Serpent 1 type robot), but the simulations carried out during the study were made using a numerical simulator such as MATLAB, and also they do not show how or which simulations were conducted. However, this paper could be replicated in another simulator such as the V-REP or Sim-Two, for example.
2. The next point that stands out as an exception (marked as **Exception 2** in Table 3), is the paper [75], Cervera, Enric, et al., “The robot programming network”. The authors present a system that allows the users to learn robotics topics in a virtual environment using a web-based laboratory with real robots or 2D/3D simulators. In this case, the system gathers tools that are fundamental for robotics learning such as learning the Robot Operating System use, including the possibility to try out in realistic and non-realistic simulators that are embedded into this web-based system.
3. Another reference that is an exception is [105], where the authors design a simulator with a realistic visualization of the head of IRYS robot. Although the simulator, made using the Unreal Engine, is realistic enough in what concern the robot motion and appearance, it is just to simulate this robot. For that reason it was categorized as an exception.
4. The last exception is the paper [106], in which the authors present an architecture for the management of a fleet of cleaning robots and, for this purpose, they design a simulator to evaluate its framework. The simulator has is called CleanSim and simulates map dirtiness; in this way, the authors can test their algorithm to improve the efficiency of the cleaning method. However, this is an exception for not being a realistic simulator based on a physics engine.

Future work could include the simulation shown in item 1 of this subsection, where the authors could replicate the modeling made in [61] with a realistic simulator. Another point to note is that, during the complete reading of the articles, some non-realistic simulators were found. However, they were discarded although they were used in educational contexts, as in the case of [105,106]; the main reason is because they were not based on a physics engine.

### 4.3. Physics Engines

Throughout the search, reading and analyzing each paper and simulator, one common point stands out, that they are built with physics engines. Table 6 shows the physics engines found and a classification in different columns depending on if they are a free or a propietary solution.

As noted in Table 6, there are many available physics engines softwares; some are paid software and others free. In [7], an evaluation among five free physics engines is presented, and the author concludes that there is no general physics engine that performs best for any given task; each has its strengths and weaknesses. Taking this previous consideration into this work, simulators based on one or more physics engines will overlap the performance of those built with only one, and that is updated repeatedly.

### 4.4. Robot Operating System-ROS

Another common feature of the simulators found during the research was the Robot Operating System (ROS). ROS is a framework for writing robot software. It has several tools, libraries and conventions that aim to simplify the task of creating complex and robust robot behavior across a wide variety of robotics platforms. This framework emerged as an alternative way to create general-purpose robot software. ROS provides standard operating system services, such as hardware abstraction and low-level device control, the implementation of commonly used features, message-passing between processes, and package management. Sets of ROS processes in execution are represented in a graph architecture where processing occurs at nodes that can receive and send messages such as multiplex sensors, control, status, planning, actuator and others. Despite the importance of reactivity and low latency in robot control, ROS itself is not a real-time operating system. For this instance, ROS is an important, free and open-source tool in the robotics field, being widely used for makers, researchers and in the industry, and it is integrated into sundry simulators such as, Gazebo, Webots, MORSE, V-REP and others (https://www.ros.org/, accessed on 8 June 2021) [35,36,43,44,49,50,52,53,75,77,87,107,108,109,110,111,112].

## 5. Conclusions and Future Work

In this paper, a systematic literature review of realistic simulators applied in an educational context was conducted in order to evaluate whether there is any simulator capable of simulating a robot prototype using realistic world physics.

By performing this systematic review, questions were answered about the found papers, providing a current state-of-the-art and a view of this research field. During the review process, 559 papers were retrieved from six different electronic databases, from which 50 relevant papers were selected and included in this review, after applying the inclusion and exclusion criteria and the quality assessment. Table 7 shows how the selected papers address the research questions asked in Section 2.1.

Therefore, by reading, analyzing and gathering data from each relevant paper and simulator, some simulators have been shown to be promising tools to be used in the educational context for some reasons that we observed.

But first, coming back to answer the research questions (RQ1, RQ2 and RQ3): considering all the simulators presented in Table 3, the frequency that was cited in the papers by the different authors, as shown in Table 4, the simulators’ features studied in Section 4.1 and Table 5, and finally, from the considerations made at the end of the previous paragraph and sections, it is possible to conclude that the simulators that can be easily applied in the educational context, are: Gazebo, Webots, SimTwo and V-REP.

The reasons for this are: firstly, the long time they have been available for use, that is, since their launch they continue to receive updates to keep up to date with the technology; The second characteristic observed was the number of platform and robots prototype variations (wheeled, legged, humanoids, drones and others) available to be used, or the possibility to add, configure and use a robot of your own in these simulators; in this way, allowing simulation in different environments, allowing a high level of abstraction with high fidelity in the simulation due to the use of physics engines. The third was the simulator’s ability to execute the simulations under one or more physics engines; this is an indicator of how realistic the simulation is. Another important feature of these simulators is that all of them have 3D vision of the robot and the environment, giving us the feeling of working with the real robot, without having it; Finally, is the capability of integrating with third party systems or protocols, for example, the integration of Robot Operating System (ROS), TCP/IP, MATLAB, LabView and others.

As future work, it could be interesting to produce a framework that provides a guideline for modelling an actuator, sensor or the entire robot in order to upload it into one of these simulators to test our own robots with different actuators or sensors, and to test them in different environments, such as a maze arena and line-following circuits.

## Figures and Tables

**Table 1:** Quality Assessment Checklist.

Quality Questions	Questions
QQ1	Is the paper based on research or is it a report based on expert opinion?
QQ2	Is there a description of the context in which the research was carried out?
QQ3	Is there a clear statement of the aims of the research?
QQ4	Was the research conducted to address the aims of the research?
QQ5	Were the simulations made during the study applied in an educational context?
QQ6	Could the simulator used in the study be applied in an educational context?
QQ7	Is the simulator used in the research able to make realistic simulations?
QQ8	Was the method used during the study described?
QQ9	Was the data analysis sufficiently rigorous?
QQ10	Is the study of value for the research field?
QQ11	Is there a clear statement of findings?

**Table 2:** Data Extraction Form.

Data Questions	Questions
DQ1	Is the simulator able to simulate robotics?
DQ2	Is the simulator able to simulate sensors?
DQ3	Is the simulator able to simulate actuators?
DQ4	What method is used for the simulator?
DQ5	Is the realistic simulation made under a mathematical–physical model?
DQ6	What is the simulator used?
DQ7	Is the simulator free to use?
DQ8	Is the simulator open-source or not?

**Table 3:** All the papers included in the SLR. In (^a^) All the selected studies about simulators. (^b^) The answer for Data Question 6 (**DQ6**), in which the answer is the name of the simulator used in the refereed study. The (^c^) column is the paper’s score in the quality assessment. Lastly, (^d^), (^e^) and (^f^) columns concern the ability to simulate robotics, sensors and actuators, addressing Data Questions (**DQ1**),(**DQ2**) and (**DQ3**), respectively.

Reference ^a^	DQ6 ^b^	Score ^c^	DQ1 ^d^	DQ2 ^e^	DQ3 ^f^
[30]	ARGoS	40	True	True	True
[31]	Sim-Two	36	True	True	True
[32]	Sim-Two	40	True	True	True
[33]	Sim-Two	40	True	True	True
[34]	Sim-Two	38	True	True	True
[35]	ROS	34	False	False	False
[36]	Gazebo	40	True	True	True
[37]	RoSoS	44	True	False	False
[38]	Sim-Two	44	True	True	True
[39]	USARSim	38	True	True	True
[40]	Breve	34	True	False	False
[41]	Sim-Two	40	True	True	True
[42]	Sim-Two	40	True	True	True
[43]	V-REP	44	True	True	True
[44]	ROS Development Studio	36	True	True	True
[45]	Gazebo	40	True	True	True
[46]	Webots	32	True	True	True
[47]	Gazebo	36	True	True	True
[48]	Simulink	34	False	True	True
[49]	V-REP	44	True	True	True
[50]	Gazebo	42	True	True	True
[51]	Sim-Two	40	True	True	True
[52]	Sim-Two	38	True	True	True
[53]	Gazebo	36	True	True	True
[54]	Sim-Two	40	True	True	True
[55]	Sim-Two	40	True	True	True
[56]	Sim-Two	40	True	True	True
[57]	Player/Stage	42	True	True	True
[58]	jmeSim	32	True	True	True
[59]	Sim-Two	44	True	True	True
[60]	Stage	40	True	True	True
[61]	**Exception 1**	34	True	True	True
[62]	Sim-Two	44	True	True	True
[63]	Sim-Two	40	True	True	True
[64]	Sim-Two	40	True	True	True
[65]	Sim-Two	32	True	True	True
[66]	Gazebo	42	True	True	True
[67]	Sim-Two	44	True	True	True
[68]	UberSim	38	True	True	True
[69]	Sim-Two	44	True	True	True
[70]	Creo	32	True	True	True
[71]	Sim-Two	42	True	True	True
[72]	Sim-Two	38	True	True	True
[73]	Sim-Two	40	True	True	True
[74]	Gazebo	42	True	True	True
[1]	MATLAB/Simulink	42	True	True	True
[75]	**Exception 2**	42	True	True	True
[76]	UberSim	40	True	True	True
[77]	V-REP	34	True	True	True
[78]	V-REP	44	True	True	True

**Table 4:** All the simulators mentioned throughout the full-read papers.

Simulators	Found in
USARSim	[30,36,39,60,75]
SimSpark	[32,33,34]
ROS Development Studio	[44]
Gazebo	[30,36,38,39,43,44,45,47,49,50,53,57,60,66,74,75,77]
Webots	[30,32,33,34,36,39,43,44,46,49,57,60,75,77]
Sim-Two	[31,32,33,38,41,42,51,52,54,55,56,59,62,64,65,67,69,72,73]
ARGoS	[30,49,60]
Stage/Player	[38,39,57,60]
MORSE	[57]
STDR	[57]
V-REP	[36,43,49,57,77,78]
RoSoS	[37]
UberSim	[32,33,34,67,76]
Breve	[40]
Teambots	[60]
MuRoSimF	[30,32,33,34]
Microsoft Robotics Studio	[32,33,36,60]
OpenHRP3	[33,77]
jmeSim	[36,58]
BOB	[38]
Khepera	[38]
Delta3D	[38]
MATLAB/Simulink	[1,48]
Swarmbot3d	[60]
Creo	[70]

**Table 5:** Simulators Features.

Simulators			
USARSim	Description	Unified System for Automation and Robot Simulator, forming the acronym USARSim, is a high-fidelity simulation of robots and environments based on the Unreal Tournament game engine. It was built upon Unreal Engine 2.0, a game engine commercially available produced by Epic Games. It has a uniform applications programmer interface, validated models, and physics-based simulation. USARSim is composed of a set of models and classes that define the simulation of robots, sensors and actuators. It has the ability to simulate several types of robots as wheeled robots, underwater vehicles, legged platforms and humanoids [39,79].	
	Features	Robot Types	Wheeled; Underwater; Legged Humanoids;
		Sensors and Actuators	Odometry; INU; Encoder; Touch; Range(Sonar, IR, Scanner); RFID Robot Camera Sound HumanMotion
		Compatibility	Player MOAST
		Engines	Unreal Engine
		Programming Languages	UnrealScript
		Other Features	Users can add new robots models, sensors and actuators; 3D Visualization; Open-Source; Free to Use;
SimSpark	Description	The SimSpark is a multi-robot simulator based on the generic components of the Spark physical multi-agent simulation system and its main application is in the Soccer Competitions. This simulator is implemented through the Open Dynamics Engine (ODE) for physically realistic dynamics simulation, allowing fast rigid body simulations, collision detection and the use of articulated body structures. It also includes a 3D visualization based on OpenGL, enabling the possibility to create scenes. The creation of new robot models it is possible using the Ruby interface in which is an interpreted language [32,33,34,80,81].	
	Features	Robot Types	Wheeled; Legged; Humanoids;
		Sensors and Actuators	Gyroscope; Motors;
		Compatibility	TCP Protocol; UDP Protocol;
		Engines	ODE OpenGL
		Programming Languages	C++ Ruby
		Other Features	Users can add new robots models, sensors and actuators; 3D Visualization; Open-Source; Free to Use;
ROS Development Studio	Description	ROS Development Studio is a web application for the simulation of robots in the cloud. This platform consists of virtual machines running in the could infrastructure provided by Amazon Web Services. The cloud platform supports two simulators, which are Gazebo and Webots [44].	
	Features	Robot Types	Not Applicable
		Sensors and Actuators	Not Applicable
		Compatibility	Not Applicable
		Engines	Not Applicable
		Programming Languages	Not Applicable
		Other Features	Not Applicable
Gazebo	Description	Gazebo simulator came up as an improvement of the Player and Stage project, and was designed to accurately reproduce the dynamic environments that a robot may encounter. It is completely open source and freely available. Its structure enables the support to simulate multi robot systems, and its major feature is the ability to easily create new robots, actuators, sensors and arbitrary objects. It was built based on the Open Dynamics Engine, and currently supports Bullet, Simbody and DART physics engines. The visualisation of Gazebo’s robots and scenes are in 3D and it was held by OpenGL and GLUT (OpenGL Utility Toolkit); at the moment it is supported by OGRE [36,47,53].	
	Features	Robot Types	Wheeled; Legged; Humanoids; Arms; Drones; Others;
		Sensors and Actuators	Several Sensors; Several Motors;
		Compatibility	Player; TCP/IP; ROS; Others;
		Engines	ODE; Bullet; Simbody; DART; OpenGL; GLUT; OGRE;
		Programming Languages	XML; C++;
		Other Features	Users can add new robots models, sensors and actuators; 3D Visualization; Open-Source; Sensors and Noise; Plugins; Robot Models; Cloud Simulation; Command Line Tools; Free to Use;
Webots	Description	Webots simulator is an open source and multi-platform desktop application applied to simulate robots. It provides a complete development environment to model, program and simulate robots. It has been designed for a professional use, and it is widely used in industry, education and research. It was developed by Cyberbotics Ltd. in 1998 and currently it is free to use. The main features about Webots is the combination of a modern graphical user interface (Qt), the ODE fork physics engine and the OpenGL rendering engine. The robot’s programming may be in C, C++, Python, Java, MATLAB and ROS. It can simulate a wide variety of robots as wheeled, industrial arms, bipeds, multi-legs, modular, automobiles, flying drones, autonomous underwater, tracked and aerospace [46,82].	
	Features	Robot Types	Two-Wheeled; Arms; Bipeds; Multi-legs; Modular; Automobiles; Drones; Autonomous Underwater; Tracked; Aerospace
		Sensors and Actuators	Several Sensors; Several Motors;
		Compatibility	ROS; TCP/IP; MATLAB; Others;
		Engines	Qt; ODE; OpenGL
		Programming Languages	C/C++; Python; Java; MATLAB; ROS;
		Other Features	Users can add new robot models, sensors and actuators; 3D Visualization; Open-Source; Asset Library of Robots, Sensors and Actuators, Objects and Materials; Free and Paid Versions;
SimTwo	Description	The SimTwo is a versatile robot simulation environment that allows the rapid test and design of differential, omnidirectional, industrial, humanoid robot and other types. It also has a set of predefined components, such as sensors and motors, where specified models are inputted. Realistic rigid body dynamics is possible thanks to the Open Dynamics Engine, and issues about the robots’ look and behaviors are written in XML format files. The visualization is in 3D, and that is provided through GLScene. SimTwo is a free software that is currently open source [38,41,48].	
	Features	Robot Types	Wheeled; Omnidirectional; Industrial; Humanoids; Others;
		Sensors and Actuators	Several Sensors; Several Motors;
		Compatibility	ROS; MATLAB; LabView; UDP Protocol; Others;
		Engines	ODE; Physics Abstraction Layer; GLScene; OpenGL;
		Programming Languages	XML; C/C++; MATLAB; Labview;
		Other Features	Asset Library of Robots, Sensors and Actuators, Objects and Materials; 3D Visualization; Free to Use;
ARGoS	Description	Autonomous Robots Go Swarming is an open source multi-robot simulator that was designed to study tools and control strategies for heterogeneous swarms of robots. It initially was released for Linux and Mac OS X, and in 2016 was updated to Windows systems. The core of its architecture is the simulated 3D space. Sensors and actuators can be implemented in a generic and efficient way, taking into account specific components instead of the complete robot. New robots can be inserted, reusing the already existing components and all the sensors/actuators, depending on those components working without modification. ARGoS supports multiple engines of different types and can run in parallel during an experiment. The 3D dynamics engine is based on the Open Dynamics Engine, and the 2D dynamics engine depends on the open-source physics engine library Chipmunk. It has either a 3D or 2D custom engine. The 3D visualization has a graphical user interface based on Qt4 and OpenGL. ARGoS and the control interface are written in C++ [30,83].	
	Features	Robot Types	Swarm Robotics
		Sensors and Actuators	Custom Sensors; Custom Motors;
		Compatibility	Not Applicable
		Engines	ODE; Chipmunk; Custom 2D and 3D Engine; Qt: OpenGL;
		Programming Languages	C++
		Other Features	Users can add new robot models, sensors and actuators; 3D Visualization; Open-Source; Free to Use;
Stage/Player	Description	Stage is a robot simulator that provides a virtual world populated by mobile robots and sensors in a two-dimensional bitmapped environment, along with various objects for the robots to sense and manipulate. It provides several sensor and actuator models, including sonar or infrared sensors, scanning laser rangefinder, color-blob tracking, fiducial tracking, bumpers, grippers and mobile robot bases with odometric or global localization. The main usage for Stage for massively multi-robot experiments, suitable for swarm robotics and other research where the behavior of large robot populations is of interest. Player is a network server for robot control. By running on the robot, it can provide a simple and clear interface for the robot’s sensors and actuators over the IP network [38,39,57,60,84].	
	Features	Robot Types	Swarm Robotics; Several Robot Models;
		Sensors and Actuators	Several Sensors Models; Several Motors;
		Compatibility	Player;
		Engines	Custom Engine
		Programming Languages	C++; TCL; Java; Python;
		Other Features	Users can add new robot models, sensors and actuators; 2D Visualization; Open-Source; Free to Use;
MORSE	Description	MORSE is a generic simulator for academic robotics. It focuses on realistic 3D simulation from small to large environments, indoor or outdoor, from one to tenths of autonomous robots and can be entirely controlled from the command-line. Simulation scenes are generated from simple Python scripts. MORSE comes with a set of standard sensors and actuators such as cameras, laser scanners, GPS, speed controllers, high-level waypoints controllers, generic joint controllers, and so forth. It also has robotic bases as quadrotors, generic four wheel vehicles, and new ones can easily be added. This simulator is based on the Blender Game Engine for rendering and the Bullet engine for physics simulation [57,85,86,87].	
	Features	Robot Types	Wheeled; Quadrotors; Others;
		Sensors and Actuators	Cameras; Laser Scanner; GPS; Speed Controllers;
		Compatibility	ROS; YARP; Pocolibs; MOOS; HLA; Mavlink;
		Engines	Blender Game Engine; Bullet
		Programming Languages	Python
		Other Features	Users can add new robot models, sensors and actuators; 3D Visualization; Open-Source; Free to Use; Asset Library of Robots, Sensors and Actuators, Objects and Materials;
STDR	Description	Simple Two Dimensional Robot Simulator as the title, the STDR simulator’s goal is to make a single robot’s or swarm’s simulation as simple as possible; as a consequence, it is not meant to be the most realistic simulator, by minimizing the needed actions to perform a experiment. STDR can function with or without a graphical environment, which allows for experiments to take place even using ssh connections [57].	
	Features	Robot Types	Not Applicable
		Sensors and Actuators	Not Applicable
		Compatibility	Not Applicable
		Engines	Not Applicable
		Programming Languages	Not Applicable
		Other Features	Not Applicable
V-REP (CoppeliaSim)	Description	The Virtual Robot Experimentation Platform or V-REP is the result of turning all requirements into a versatile and scalable simulation framework. According to its website https://www.coppeliarobotics.com/, accessed on 8 June 2021, V-REP was discontinued in November 26 2019, becoming CoppeliaSim Robot Simulator and owning an integrated development environment based on a distributed control architecture, which means that each object/model can be individually controlled via an embedded script such as ROS, BlueZero node, a remote API client or a custom solution. CoppeliaSim is very versatile and ideal for multi-robot applications. Controllers can be written in C/C++, Python, Java, Lua, Matlab or Octave. It can be applied for fast algorithm development, factory automation simulations, fast prototyping and verification, robotics related education, remote monitoring, safety double-checking, as a digital twin, and much more [43,49,77,78,88].	
	Features	Robot Types	multi-robots
		Sensors and Actuators	multi-sensors
		Compatibility	ROS; BlueZero; LabView; TCP/IP;
		Engines	Bullet; ODE; Vortex; Newton;
		Programming Languages	C/C++; Lua; Java; Python; LabView; MATLAB; Octave;
		Other Features	Asset Library of Robots, Sensors and Actuators, Objects and Materials; 3D Visualization; Free and Paid Versions;
RoSoS	Description	Robot Soccer Simulator is a simulator platform that was built and programmed using Processing language, and considers two guidelines: 1. the program for a virtual robot should be similar to the program of a real robot; and 2. users should have the possibility to change the simulator operation, such as physics and game rules, and also to personalize their robots. It is free, open-source and runs on Windows, Mac OS and Linux [37].	
	Features	Robot Types	Soccer Robots
		Sensors and Actuators	Ball Sensor; Compass Sensor; Ultrassonic Sensor;
		Compatibility	Not Applicable
		Engines	Custom Engine
		Programming Languages	C++; Java;
		Other Features	Noise and Imprecisions; 2D Visualization; Open-Source; Free to Use;
UberSim	Description	A Multi-Robot Simulator for Robot Soccer was created as a free and open-source simulator project, with the objective of being a robot soccer simulation engine with high-fidelity dynamics and collision models and extensible robot classes, built based on the Open Dynamics Engine [76]. A search was made in order to obtain more information about the ÜberSim simulator but no further information was found, even its website was off-line. Thus, it was considered discontinued.	
	Features	Robot Types	Multi-Agent Systems; Artifial Life;
		Sensors and Actuators	Not Applicable
		Compatibility	Not Applicable
		Engines	OpenGL;
		Programming Languages	Python; Steve;
		Other Features	3D Visualization; Free to Use; Open-Source;
Breve	Description	Breve is a free, open-source software package that makes it easy to build 3D simulations of multi-agent systems and artificial life. Using Python, or using a simple scripting language called steve, you can define the behaviors of agents in a 3D world and observe how they interact. Breve includes physical simulation and collision detection; it is possible to simulate realistic creatures and to use an OpenGL display engine to visualize simulated worlds. However, according to its website http://www.spiderland.org/s/, accessed on 8 June 2021, the project has not been maintained since 2009, but it is still available for download for Windows, Linux and Mac OS [40].	
	Features	Robot Types	Wheeled; Legged; Humanoids; Arms; Drones; Others;
		Sensors and Actuators	Several Sensors; Several Motors;
		Compatibility	Player; TCP/IP; ROS; Others;
		Engines	ODE; Bullet; Simbody; DART; OpenGL; GLUT; OGRE;
		Programming Languages	XML; C++;
		Other Features	Users can add new robots models, sensors and actuators; 3D Visualization; Open-Source; Sensors and Noise; Plugins; Robot Models; Cloud Simulation; Command Line Tools; Free to Use;
TeamBots	Description	TeamBots is a 2D Java-based simulator for multi-agent mobile robotics research. The simulation environment and the robots are written mostly in Java. It supports multiple heterogeneous robot hardware running heterogeneous control systems. Complex (or simple) experimental environments can be designed with walls, roads, opponent robots and circular obstacles. All of these objects may be included in a simulation by editing an easily understandable human-readable description file. TeamBots runs under Windows, Linux and MacOS. The search for information about this simulator was not an easy task and since the latest version of this platform was updated in April 2000, it can be considered discontinued [60].	
	Features	Robot Types	Nomad 150; Cye Robot;
		Sensors and Actuators	Not Applicable
		Compatibility	Not Applicable
		Engines	Not Applicable
		Programming Languages	Java
		Other Features	2D Visualization; Free to Use; Open-Source;
MuRoSimF	Description	The Multi-Robot-Simulation-Framework (MuRoSimF) provides an easy way to generate interactive simulations for the motion and sensing capabilities of wheeled, biped and multi-legged robots. Unlike most existing robot simulation packages, MuRoSimF is not limited to predefined simulation algorithms (e.g., for dynamics or sensor simulation). Instead of this, it provides a flexible and modular way to combine simulation models of the robots and algorithms. By this, it is possible to generate simulations, which are scalable in their level of physical accuracy, level of detail and computational complexity, thus enabling the user to create simulations that are adequate to a given task. Using MuRoSimF, several simulations for mobile robots including wheeled, biped and four-legged devices have been created. MuRoSimF provides a range of exchangeable algorithms for motion simulation (kinematic, simplified dynamics, full multi-body-system dynamics), collision detection and sensor simulation (including cameras, laser range finders and inertial sensors). More modules, for example, for detailed simulation of servo motors can be added to the framework easily [89].	
	Features	Robot Types	Wheeled; Bipeds; Multi-Legged
		Sensors and Actuators	Cameras; Laser Range Finders; Inertial Sensors;
		Compatibility	Not Applicable
		Engines	OpenGL; Custom Dynamics Engine;
		Programming Languages	C++
		Other Features	2D Visualization; 3D Visualization; Free to Use; Open-Source;
Microsoft Robotics Studio	Description	Microsoft Robotics Studio is an environment for robot control and simulation. It was designed for academic, hobbyist and commercial developers, and it also contains a wide variety of robot hardware. A point to note about this simulator is that it is only available on the Windows operational system. The main features include the Microsoft Visual Programming Language for creating and debugging robot applications and 3D simulation with access to the robot’s sensors and actuators. Unfortunately, this simulator was discontinued in 2012 [90,91,92].	
	Features	Robot Types	multi-robots
		Sensors and Actuators	Several Sensors; Several Motors;
		Compatibility	OpenCV; CodePlex;
		Engines	NVIDIA PhysX
		Programming Languages	C#; Microsoft Visual Programming Language;
		Other Features	3D Visualization; Free and Paid Versions; Discontuated;
OpenHRP3	Description	Open Architecture Human-centered Robotics Platform version 3 is a platform for robot simulations and software developments, and it is mainly applied for humanoid robot simulations. It allows users to check out a robot model and control program by dynamics simulation. The simulator has a custom dynamics engine and graphical interface. The last version was released in 2012 [93,94].	
	Features	Robot Types	Humanoids
		Sensors and Actuators	Several Sensors; Several Motors;
		Compatibility	Not Applicable
		Engines	Custom Dynamics Engine; Custom Graphical Engine;
		Programming Languages	C++
		Other Features	3D Visualitation; Free to Use; Open-Source;
jmeSim	Description	jmeSim is an open source, multi-robot platform; it provides high graphical and physical fidelity and also supports ROS integration. It was built on the jMonkey Engine3 game engine. The physics dynamics simulation is performed by jBullet, a Java port of the Bullet Physics Engine Library. The jmeSim offers some environment and robot models, such as the wheeled rescue robot for example, and also provides an array of sensors [58].	
	Features	Robot Types	multi-robots
		Sensors and Actuators	Several Sensors; Several Motors;
		Compatibility	ROS
		Engines	jBullet; jMonkey;
		Programming Languages	Java
		Other Features	3D Visualization; Free to Use; Open-Source;
Khepera	Description	Khepera Simulator is a freeware public domain software written by Oliver Michel, and was designed to simulate the Khepera robot. This package allows the programmer to write control algorithms using C/C++ language. The simulator runs on Unix operational system and it has X11 as graphical interface. This simulator features the ability to drive a real Khepera robot, then the outcome of the simulation test can be easily transferred to a real Khepera robot. No further information about this simulator was found and its website, presented in [95], is not available.	
	Features	Robot Types	Khepera Robots
		Sensors and Actuators	Not Applicable
		Compatibility	Not Applicable
		Engines	X11
		Programming Languages	C/C++
		Other Features	2D Visualization; Free to Use; Discontinuated;
Delta3D	Description	The simulator Delta3D is an open source game and simulation engine built for military training. Delta3d is a widely used, community-supported, open-source game and simulation engine. Delta3d is appropriate for a wide variety of uses including training, education, visualization and entertainment. Delta3d is unique because it offers features specifically suited to the Modeling, Simulation and DoD communities, such as the High Level Architecture (HLA), After Action Review (AAR), large scale terrain support and SCORM Learning Management System (LMS) integration. It has a modular design integrating other engines such as Open Scene Graph, Open Dynamics Engine, Character Animation Library and OpenAL. The renders engine uses the Open Graphics Library. The last version released was on September 29 2014 and it is held on Github [96,97].	
	Features	Robot Types	Not Applicable
		Sensors and Actuators	Not Applicable
		Compatibility	Not Applicable
		Engines	Open Scene Graph; ODE; Character Animation Library; OpenAL; Open Graphics Library;
		Programming Languages	C++
		Other Features	3D Visualization; Free to Use; Open-Source; Military Purpose;
MATLAB/ Simulink	Description	MATLAB is a powerful general software that aids scientists, researchers and companies in several engineering/science areas, such as control systems, deep learning, image processing and computer vision, machine learning, predictive maintenance, robotics, signal processing, test and measurement of data and wireless communications. Simulink is a block diagram environment for multi domain simulation and Model-Based Design. It supports system-level design, simulation, automatic code generation and continuous testing and verification of embedded systems. Simulink provides a graphical editor, customized block libraries, and solvers for modeling and simulating dynamic systems. It is integrated with MATLAB, enabling the incorporation of MATLAB algorithms into models and the export of simulation results to MATLAB for further analysis. In the robotics field, MATLAB with Simulink is one of the most used platforms for the modeling and simulation of several systems. Many features can be added for the simulation, as well as the simulation of dynamics and graphical modelling, with the possibility of working in real time. To accomplish these features, it needs to be added that it is called “toolboxes” [1,98,99,100,101].	
	Features	Robot Types	Any Robot Type
		Sensors and Actuators	Any Type
		Compatibility	Several Possibilities
		Engines	Custom Engine
		Programming Languages	Several Languages Supported
		Other Features	2D and 3D Visualization; Paid;
Swarmbot3D	Description	Swambot3d was designed for predicting the 3D kinematics and dynamics of a single s-bot in a swarm-bot. The main characteristics of this simulation environment are: 3D dynamics, compatibility with the s-bot’s hardware and software, interactive control, multi-level models and swarm handling, and it was built using the Vortex physics engine. The simulation models of the environment and robots are defined in an external test file written in XML format. A search was evaluated to find this simulator on the internet and no results were found [102,103,104].	
	Features	Robot Types	s-bot
		Sensors and Actuators	Available under Modelling
		Compatibility	Not Applicable
		Engines	Vortex
		Programming Languages	XML
		Other Features	Not Applicable
Creo	Description	In [70] the authors use a different approach to simulate a humanoid robot using its virtual twin. The robot modelling was made using the Creo software from PTC Inc., and the Creo is not a simulator, but the presented approach is an interesting and different way to simulate a robot, differing from the previously shown simulators. Creo is a 3D computer-aided design software for product development with scalable range. Creo has breakthrough innovations in the areas of generative design, real-time simulation, multi-body design, additive manufacturing, and other features.	
	Features	Robot Types	Not Applicable
		Sensors and Actuators	Not Applicable
		Compatibility	Not Applicable
		Engines	Not Applicable
		Programming Languages	Not Applicable
		Other Features	Not Applicable

**Table 6:** Physics Engines.

Free/Open-Source	Proprietary
Box2D	AGX Multiphysics
Bullet	Algodoo
Cannon.js	Digital Molecular Matter
Chipmunk	Chipmunk
Newton Game Dynamics	Euphoria
Open Dynamics Engine	Havok
OPAL	Reactor
Physics Abstraction Layer	Vortex
PhysX	
PhyZ	
Project Chrono	
Siconos	
Simulation Open Framework Architecture	

**Table 7:** Selected papers that address the Research Questions.

Research Questions	Selected Papers
1: In the context of educational robotics, are there any realistic simulators capable of simulating any robot prototype?	[30,32,33,36,37,38] [41,42,43,45,49,50] [51,54,55,56,57,59] [60,62,63,64,66,67] [69,71,73,74,75,76,78]
2: Are these simulators capable of simulating the robot’s sensores and/or actuators?	[30,31,32,33,34,35] [36,38,39,41,42,43] [44,45,46,47,48,49] [50,51,52,53,54,55] [56,57,58,59,60,61] [62,63,64,65,66,67] [68,69,70,71,72,73] [1,74,75,76,77,78]
3: Is such simulation based on physical motors?	[30,31,32,33,34,36] [37,38,40,41,42,43] [46,47,48,49,50,51] [53,55,56,58,60,61] [62,63,64,65,67,68] [1,69,72,73,74,76,77]

## Abbreviations

The following abbreviations are used in this manuscript:

SLR	Systematic Literature Review
PICOC	Population, Intervention, Comparison, Outcomes, Context
PRISMA	Preferred Reporting Items for Systematic Reviews and Meta-Analyses
RQ	Research Question
IC	Inclusion Criteria
EC	Exclusion Criteria
QQ	Quality Question
DQ	Data Question
ROS	Robot Operating System

## Appendix A. Data Repository

https://github.com/caioorafael/Systematic-Literature-Review-of-Realistic-Simulators-to-be-applied-in-Educational-Context.git, accessed on 8 June 2021.

## References

[1] Žlajpah L. (2008). Simulation in robotics. Math. Comput. Simul..

[2] Reckhaus M., Hochgeschwender N., Paulus J., Shakhimardanov A., Kraetzschmar G.K. (2010). An overview about simulation and emulation in robotics. Proc. Simpar.

[3] Williams E.J., Ülgen O.M., Bangsow S. (2012). Simulation Applications in the Automotive Industry. Use Cases of Discrete Event Simulation.

[4] Xu J., Huang E., Hsieh L., Lee L.H., Jia Q.-S., Chen C.-H. (2016). Simulation optimization in the era of Industrial 4.0 and the Industrial Internet. J. Simul..

[5] Currie C.S., Fowler J.W., Kotiadis K., Monks T., Onggo B.S., Robertson D.A., Tako A.A. (2020). How simulation modelling can help reduce the impact of COVID-19. J. Simul..

[6] Boeing A., Bräunl T. Evaluation of real-time physics simulation systems. Proceedings of the 5th International Conference on Computer Graphics and Interactive Techniques in Australia and Southeast Asia.

[7] Hummel J., Wolff R., Stein T., Gerndt A., Kuhlen T. (2012). An evaluation of open source physics engines for use in virtual reality assembly simulations. International Symposium on Visual Computing.

[8] Bourg D.M., Bywalec B. (2013). Physics for Game Developers: Science, Math, and Code for Realistic Effects.

[9] Millington I. (2007). Game Physics Engine Development.

[10] Ferrada-Ferrada C., Carrillo-Rosúa J., Díaz-Levicoy D., Silva-Díaz F. (2020). Robotics from STEM areas in Primary School: A Systematic Review. Educ. Knowl. Soc..

[11] Conde M.Á., Rodríguez-Sedano F.J., Fernández-Llamas C., Gonçalves J., Lima J., García-Peñalvo F.J. (2021). Fostering STEAM through Challenge Based Learning, Robotics and Physical Devices: A systematic mapping literature review. Comput. Appl. Eng. Educ..

[12] García-Holgado A., Marcos-Pablos S., García-Peñalvo F.J. (2020). Guidelines for performing Systematic Research Projects Reviews. Int. J. Interact. Multimed. Artif. Intell..

[13] Kitchenham B. (2004). Procedures for Performing Systematic Reviews.

[14] Kitchenham B., Charters S. (2007). Guidelines for Performing Systematic Literature Reviews in Software Engineering.

[15] Kitchenham B.A., Budgen D., Brereton P. (2015). Evidence-Based Software Engineering and Systematic Reviews.

[16] Dyba T., Dingsoyr T., Hanssen G.K. Applying systematic reviews to diverse study types: An experience report. Proceedings of the First International Symposium on Empirical Software Engineering and Measurement (ESEM 2007).

[17] Cooper H.M. (1998). Synthesizing Research: A Guide for Literature Reviews.

[18] Orwin R.G., Cooper I.H., Hedges L.V. (1994). The Handbook of Research Synthesis.

[19] Dybå T., Kampenes V.B., Sjøberg D.I.K. (2006). A Systematic Review of Statistical Power in Software Engineering Experiments. Inf. Softw. Technol..

[20] Higgins J.P.T., Green S. (2005). Cochrane Handbook for Systematic Reviews of Interventions 4.2.5 [updated May 2005]. The Cochrane Library.

[21] Mulrow C., Cook D. (1998). Systematic Reviews: Synthesis of Best Evidence for Health Care Decisions.

[22] Petticrew M., Roberts H. (2006). Systematic Reviews in the Social Sciences: A Practical Guide.

[23] Popay J., Roberts H., Sowden A., Petticrew M., Britten N., Arai L., Roen K., Rodgers M. (2005). Developing guidance on the conduct of narrative synthesis in systematic reviews. J. Epidemiol. Community Health.

[24] Jpt Chh G.S. (2011). Cochrane Handbook for Systematic Reviews of Interventions Version 5.1. 0.

[25] La A.H., Szabo I., Le Brun L., Owen I., Fletcher G., Hill M. (2011). An evidence-based approach to scoping reviews. Electron. J. Inf. Syst. Eval..

[26] Ferreras-Fernández T., Martín-Rodero H., García-Peñalvo F.J., Merlo-Vega J.A. The systematic review of literature in LIS: An approach. Proceedings of the Fourth International Conference on Technological Ecosystems for Enhancing Multiculturality.

[27] Rodero H.M. (2014). La búSqueda Bibliográfica, Pilar Fundamental de la Medicina Basada en la Evidencia: Evaluación Multivariante en las Enfermedades Nutricionales y Metabólicas. Ph.D. Thesis.

[28] Moher D., Altman D.G., Liberati A., Tetzlaff J. (2011). PRISMA statement. Epidemiology.

[29] Moher D., Liberati A., Tetzlaff J., Altman D.G., Prisma Group (2009). Preferred reporting items for systematic reviews and meta-analyses: The PRISMA statement. PLoS Med..

[30] Pinciroli C., Trianni V., O’Grady R., Pini G., Brutschy A., Brambilla M., Mathews N., Ferrante E., Di Caro G., Ducatelle F. ARGoS: A modular, multi-engine simulator for heterogeneous swarm robotics. Proceedings of the 2011 IEEE/RSJ International Conference on Intelligent Robots and Systems.

[31] Gonçalves J., Lima J., Costa P.G. (2015). DC motors modeling resorting to a simple setup and estimation procedure. CONTROLO’2014–Proceedings of the 11th Portuguese Conference on Automatic Control.

[32] Lima J.L., Goncalves J.C., Costa P.G., Moreira A.P. (2010). Humanoid low-level controller development based on a realistic simulation. Int. J. Humanoid Robot..

[33] Lima J., Gonçalves J., Costa P., Moreira A. Humanoid realistic simulator: The servomotor joint modeling. Proceedings of the 6th International Conference on Informatics in Control, Automation and Robotics.

[34] Lima J., Gonçalves J., Costa P., Moreira A. (2008). Humanoid robot simulator: A realistic dynamics approach. CONTROLO 2008-The 8th Portuguese Conference on Automatic Control.

[35] Quigley M., Conley K., Gerkey B., Faust J., Foote T., Leibs J., Wheeler R., Ng A.Y. (2009). ROS: An open-source Robot Operating System. ICRA Workshop Open Source Softw..

[36] Afanasyev I., Sagitov A., Magid E. ROS-based SLAM for a Gazebo-simulated mobile robot in image-based 3D model of indoor environment. Proceedings of the International Conference on Advanced Concepts for Intelligent Vision Systems.

[37] Martins F.N., Gomes I.S., Santos C.R. (2016). RoSoS-A free and open-source robot soccer simulator for educational robotics. Robotics.

[38] Paulo C., José G., José L., Paulo M. (2011). Simtwo realistic simulator: A tool for the development and validation of robot software. Theory Appl. Math. Comput. Sci..

[39] Carpin S., Lewis M., Wang J., Balakirsky S., Scrapper C. USARSim: A robot simulator for research and education. Proceedings of the 2007 IEEE International Conference on Robotics and Automation.

[40] Klein J., Spector L. (2009). 3d multi-agent simulations in the breve simulation environment. Artificial Life Models in Software.

[41] Eckert L., Piardi L., Lima J., Costa P., Valente A., Nakano A. (2019). 3D Simulator Based on SimTwo to Evaluate Algorithms in Micromouse Competition. World Conference on Information Systems and Technologies.

[42] Piardi L., Eckert L., Lima J., Costat P., Valente A., Nakano A. 3D simulator with hardware-in-the-loop capability for the micromouse competition. Proceedings of the 2019 IEEE International Conference on Autonomous Robot Systems and Competitions (ICARSC).

[43] Farias G., Fabregas E., Peralta E., Torres E., Dormido S. (2017). A Khepera IV library for robotic control education using V-REP. IFAC-PapersOnLine.

[44] Cervera E., Casañ G., Tellez R. (2017). Cloud Simulations for RoboCup. Robot World Cup.

[45] Ferrein A., Maier C., Mühlbacher C., Niemueller T., Steinbauer G., Vassos S. Controlling logistics robots with the action-based language YAGI. Proceedings of the International Conference on Intelligent Robotics and Applications.

[46] Michel O. (2004). Cyberbotics Ltd. Webots™: Professional mobile robot simulation. Int. J. Adv. Robot. Syst..

[47] Koenig N., Howard A. Design and use paradigms for gazebo, an open-source multi-robot simulator. Proceedings of the 2004 IEEE/RSJ International Conference on Intelligent Robots and Systems (IROS) (IEEE Cat. No. 04CH37566).

[48] Denisov A., Budkov V., Mikhalchenko D. Designing simulation model of humanoid robot to study servo control system. Proceedings of the International Conference on Interactive Collaborative Robotics, ICR 2016.

[49] Peralta E., Fabregas E., Farias G., Vargas H., Dormido S. (2016). Development of a Khepera IV Library for the V-REP Simulator. IFAC-PapersOnLine.

[50] Chebotareva E., Gavrilova L. Educational Mobile Robotics Project “ROS-Controlled Balancing Robot” Based on Arduino and Raspberry Pi. Proceedings of the 2019 12th International Conference on Developments in eSystems Engineering (DeSE).

[51] Gonçalves J., Lima J., Malheiros P., Costa P. (2010). Fostering advances in mechatronics and robotics resorting to simulation. IFAC Proc. Vol..

[52] Pinho T., Moreira A.P., Boaventura-Cunha J. (2015). Framework using ROS and SimTwo simulator for realistic test of mobile robot controllers. CONTROLO’2014–Proceedings of the 11th Portuguese Conference on Automatic Control.

[53] Shimchik I., Sagitov A., Afanasyev I., Matsuno F., Magid E. (2016). Golf cart prototype development and navigation simulation using ROS and Gazebo. MATEC Web of Conferences.

[54] Lima J., Costa P., Brito T., Piardi L. Hardware-in-the-loop simulation approach for the Robot at Factory Lite competition proposal. Proceedings of the 2019 IEEE International Conference on Autonomous Robot Systems and Competitions (ICARSC).

[55] Lima J., Gonçalves J., Costa P., Moreira A. Humanoid robot gait planning resorting to an adaptive simulated annealing algorithm. Proceedings of the 10th Conference on Autonomous Robot Systems and Competitions.

[56] Lima J.L., Gonçalves J.C., Costa P.G., Moreira A.P. Humanoid robot simulation with a joint trajectory optimized controller. Proceedings of the 2008 IEEE International Conference on Emerging Technologies and Factory Automation.

[57] Ferreira N.F., Araujo A., Couceiro M.S., Portugal D. (2020). Intensive summer course in robotics–Robotcraft. Appl. Comput. Inform..

[58] Haber A., McGill M., Sammut C. Jmesim: An open source, multi platform robotics simulator. Proceedings of the Australasian Conference on Robotics and Automation.

[59] Costa P.J., Moreira N., Campos D., Gonçalves J., Lima J., Costa P.L. (2016). Localization and navigation of an omnidirectional mobile robot: the robot@ factory case study. IEEE Rev. Iberoam. Tecnol. Del Aprendiz..

[60] Vaughan R. (2008). Massively multi-robot simulation in stage. Swarm Intell..

[61] Das M.T., Dülger L.C. (2005). Mathematical modelling, simulation and experimental verification of a scara robot. Simul. Model. Pract. Theory.

[62] Campos D., Santos J., Gonçalves J., Costa P. (2016). Modeling and simulation of a hacked neato xv-11 laser scanner. Robot 2015: Second Iberian Robotics Conference.

[63] Lima J., Gonçalves J., Costa P.J., Moreira A.P. (2013). Modeling and simulation of a laser scanner sensor: An industrial application case study. Advances in Sustainable and Competitive Manufacturing Systems.

[64] Gonçalves J., Lima J., Costa P.J., Moreira A.P. (2013). Modeling and simulation of the emg30 geared motor with encoder resorting to simtwo: The official robot@ factory simulator. Advances in Sustainable and Competitive Manufacturing Systems.

[65] Lima J., Gonçalves J., Costa P.J. (2015). Modeling of a low cost laser scanner sensor. CONTROLO’2014–Proceedings of the 11th Portuguese Conference on Automatic Control.

[66] Vega J., Cañas J.M. (2018). PiBot: An open low-cost robotic platform with camera for STEM education. Electronics.

[67] Gonçalves J., Silva M., Costa P., Sousa A. Proposal of a low cost educational mobile robot experiment: An approach based on hardware and simulation. Proceedings of the 6th Internation Conference on Robotics on Education.

[68] Lima J., Gonçalves J., Costa P., Moreira A. Realistic behaviour simulation of a humanoid robot. Proceedings of the 8th Conference on Autonomous Robot Systems and Competitions.

[69] Gonçalves J., Lima J., Malheiros P., Costa P. Realistic simulation of a lego mindstorms nxt based robot. Proceedings of the 2009 IEEE Control Applications, (CCA) & Intelligent Control.

[70] Verner I., Cuperman D., Fang A., Reitman M., Romm T., Balikin G. (2018). Robot online learning through digital twin experiments: A weightlifting project. Online Engineering & Internet of Things.

[71] Braun J., Fernes L.A., Moya T., Oliveira V., Brito T., Lima J., Costa P. Robot@ factory lite: An educational approach for the competition with simulated and real environment. Proceedings of the Iberian Robotics Conference.

[72] Gonçalves J., Lima J., Oliveira H., Costa P. Sensor and actuator modeling of a realistic wheeled mobile robot simulator. Proceedings of the 2008 IEEE International Conference on Emerging Technologies and Factory Automation.

[73] Gonçalves J., Lima J., Malheiros P., Costa P. Sensor and actuator stochastic modeling of the Lego Mindstorms NXT educational Kit. Proceedings of the 10th Conference on Mobile Robots and Competitions.

[74] Zwilling F., Niemueller T., Lakemeyer G. (2014). Simulation for the RoboCup logistics league with real-world environment agency and multi-level abstraction. Robot Soccer World Cup.

[75] Cervera E., Martinet P., Marin R., Moughlbay A.A., Del Pobil A.P., Alemany J., Esteller R., Casañ G. (2016). The robot programming network. J. Intell. Robot. Syst..

[76] Browning B., Tryzelaar E. Übersim: a multi-robot simulator for robot soccer. Proceedings of the Second International Joint Conference on Autonomous Agents and Multiagent Systems.

[77] Rohmer E., Singh S.P., Freese M. V-REP: A versatile and scalable robot simulation framework. Proceedings of the 2013 IEEE/RSJ International Conference on Intelligent Robots and Systems.

[78] Gawryszewski M., Kmiecik P., Granosik G. (2017). V-REP and LabVIEW in the Service of Education. Robotics in Education.

[79] Petry M., Moreira A.P., Reis L.P., Rossetti R. Intelligent wheelchair simulation: Requirements and architectural issues. Proceedings of the 11th International Conference on Mobile Robotics and Competitions.

[80] Boedecker J., Asada M. (2008). Simspark–concepts and application in the robocup 3d soccer simulation league. Auton. Robot..

[81] Xu Y., Vatankhah H. (2013). Simspark: An open source robot simulator developed by the robocup community. Robot Soccer World Cup.

[82] Michel O. (1998). Webots: Symbiosis between virtual and real mobile robots. International Conference on Virtual Worlds.

[83] Pinciroli C., Trianni V., O’Grady R., Pini G., Brutschy A., Brambilla M., Mathews N., Ferrante E., Di Caro G., Ducatelle F. (2012). ARGoS: A modular, parallel, multi-engine simulator for multi-robot systems. Swarm Intell..

[84] Gerkey B., Vaughan R.T., Howard A. The player/stage project: Tools for multi-robot and distributed sensor systems. Proceedings of the 11th International Conference on Advanced Robotics.

[85] Lemaignan S., Echeverria G., Karg M., Mainprice J., Kirsch A., Alami R. Human-robot interaction in the MORSE simulator. Proceedings of the Seventh Annual ACM/IEEE International Conference on Human-Robot Interaction.

[86] Echeverria G., Lassabe N., Degroote A., Lemaignan S. Modular open robots simulation engine: Morse. Proceedings of the 2011 IEEE International Conference on Robotics and Automation.

[87] Noori F.M., Portugal D., Rocha R.P., Couceiro M.S. On 3D simulators for multi-robot systems in ROS: MORSE or Gazebo?. Proceedings of the 2017 IEEE International Symposium on Safety, Security and Rescue Robotics (SSRR).

[88] Freese M., Singh S., Ozaki F., Matsuhira N. Virtual robot experimentation platform v-rep: A versatile 3d robot simulator. Proceedings of the International Conference on Simulation, Modeling, and Programming for Autonomous Robots.

[89] Friedmann M. (2010). Simulation of Autonomous Robot Teams with Adaptable Levels of Abstraction. Ph.D. Thesis.

[90] Jackson J. (2007). Microsoft robotics studio: A technical introduction. IEEE Robot. Autom. Mag..

[91] Cepeda J.S., Chaimowicz L., Soto R. Exploring Microsoft Robotics Studio as a mechanism for service-oriented robotics. Proceedings of the 2010 Latin American Robotics Symposium and Intelligent Robotics Meeting.

[92] Workman K., Elzer S. (2009). Utilizing Microsoft robotics studio in undergraduate robotics. J. Comput. Sci. Coll..

[93] Kanehiro F., Hirukawa H., Kajita S. (2004). Openhrp: Open architecture humanoid robotics platform. Int. J. Robot. Res..

[94] Cisneros R., Yoshida E., Yokoi K. Ball dynamics simulation on openhrp3. Proceedings of the 2012 IEEE International Conference on Robotics and Biomimetics (ROBIO).

[95] Michel O. (1996). Khepera Simulator Version 2.0, User Manual. Université de Nice–Sophia Antipolis.

[96] McDowell P., Darken R., Sullivan J., Johnson E. (2006). Delta3D: A complete open source game and simulation engine for building military training systems. J. Def. Model. Simul..

[97] Darken R., McDowell P., Johnson E. (2005). Projects in VR: The Delta3D open source game engine. IEEE Comput. Graph. Appl..

[98] Corke P.I. (1996). A robotics toolbox for MATLAB. IEEE Robot. Autom. Mag..

[99] Corke P.I. A computer tool for simulation and analysis: The Robotics Toolbox for MATLAB. Proceedings of the Australian Conference on Robotics Association.

[100] Toz M., Kucuk S. (2010). Dynamics simulation toolbox for industrial robot manipulators. Comput. Appl. Eng. Educ..

[101] Karakaya S., Kucukyildiz G., Ocak H. (2017). A new mobile robot toolbox for MATLAB. J. Intell. Robot. Syst..

[102] Mondada F., Pettinaro G.C., Guignard A., Kwee I.W., Floreano D., Deneubourg J.L., Nolfi S., Gambardella L.M., Dorigo M. (2004). SWARM-BOT: A new distributed robotic concept. Auton. Robot..

[103] Pettinaro G.C., Kwee I.W., Gambardella L.M. (2003). Definition, Implementation, and Calibration of the Swarmbot3d Simulator.

[104] Pettinaro G.C., Kwee I.W., Gambardella L.M. (2003). Swarmbot3D User Manual. https://repository.supsi.ch/5558/1/IDSIA-22-03.pdf.

[105] Dąbek P., Trojnacki M., Jaroszek P., Zawieska K. Concept, Physical Design and Simulator of IRYS Social Robot Head. Proceedings of the International Conference Mechatronics.

[106] Costa H., Tavares P., Santos J., Rio V., Sousa A. Simulation of a System Architecture for Cooperative Robotic Cleaning. Proceedings of the Robot 2015: Second Iberian Robotics Conference.

[107] Couceiro M.S., Araújo A.G., Tatarian K., Ferreira N.M. (2018). RobotCraft: The first international collective internship for advanced robotics training. International Conference on Robotics and Education RiE 2017.

[108] Conte G., Scaradozzi D., Mannocchi D., Raspa P., Panebianco L., Screpanti L. (2018). Development and experimental tests of a ROS multi-agent structure for autonomous surface vehicles. J. Intell. Robot. Syst..

[109] Araújo A., Portugal D., Couceiro M.S., Rocha R.P. (2015). Integrating Arduino-based educational mobile robots in ROS. J. Intell. Robot. Syst..

[110] Tatarian K., Pereira S., Couceiro M.S., Portugal D. (2018). Tailoring a ROS educational programming language architecture. International Conference on Robotics and Education RiE.

[111] Koubâa A. (2017). Robot Operating System (ROS).

[112] Quigley M., Gerkey B., Smart W.D. (2015). Programming Robots with ROS: A Practical Introduction to the Robot Operating System.

